# INSARA: a new method for the analysis and visualization of Structure-Activity-Relationships

**DOI:** 10.1186/1758-2946-4-S1-P44

**Published:** 2012-05-01

**Authors:** Sabrina Wollenhaupt, Knut Baumann

**Affiliations:** 1 Institut für Medizinische und Pharmazeutische Chemie, Technische Universität Braunschweig, Beethovenstr. 55, D-38106 Braunschweig, Germany

## 

Due to the rapid progress in combinatorial chemistry and (high-throughput-) screening, the organization and mining of the large amount of produced data becomes an increasingly important task in the modern drug discovery process. Herein, one particular challenge is the recognition of SAR-patterns e.g. for the selection of promising compounds for further analysis or lead optimization.

To support the medicinal chemist in doing this job a readily interpretable concept is required. Most published approaches addressing this problem (e.g. SARANEA [1]) use fingerprint similarity for the analysis of molecular relationships. Yet, a promising alternative and more intuitive way of comparing similarity is the maximum common substructure (MCS), the largest substructure in common between two molecules. Since computing the MCS is very demanding, it is usually not applicable to large data sets.

To circumvent this and other drawbacks (e.g. the exact match or incomplete ring problems) our own in-house strategy **INSARA** (**i**ntuitive **n**etworks for **S**tructure-**A**ctivity-**R**elationships **a**nalysis) employs reduced graphs (RG) instead of the original molecules in order to reduce the complexity of the problem to a manageable size.

The advantage of RG is that only pharmacophoric features and functional units represented by a few pseudoatoms have to be compared [2]. Iterative super- and substructure searches and MCS calculations subsequently lead in an unsupervised manner to SAR networks. When associated with bioactivity data the networks can be used for SAR analysis. While focussing on pharmacophoric properties a more general overview about the similarities within the active set is expected. For initial performance evaluation two targets with well-known SARs (ACE and COX 2) were chosen.
